# Short- to Mid-Term Outcomes in Arthroscopic Debridement of the Knee: A Prospective Case Series

**DOI:** 10.7759/cureus.32349

**Published:** 2022-12-09

**Authors:** Nachappa Sivanesan Uthraraj, Ravali Suguru, Fitzgerald Anazor, Ali Hussain, Anand B Divekar, Kumar Gaddam Raddy, Raj Shrivastava, Jai Relwani

**Affiliations:** 1 Trauma and Orthopaedics, William Harvey Hospital, Ashford, GBR; 2 Trauma and Orthopaedics, Gandhi Medical College and Hospital, Hyderabad, IND; 3 Trauma and Orthopaedics, Chalmeda Anand Rao Institute of Medical Sciences, Karimnagar, IND

**Keywords:** oxford knee score, debridement and lavage, knee joint, arthroscopy, osteoarthritis

## Abstract

Introduction

Osteoarthritis of the knee is a common debilitating disease in the elderly population. There are many treatment options available including physiotherapy, analgesics, steroid injections, arthroscopic debridement, high tibial osteotomy (HTO) and arthroplasty. Arthroscopic debridement is beneficial when patients are chosen with certain characteristics. This is a prospective case series where we have correlated the patient and disease characteristics, with the pre- and post-operative Oxford Knee Scores (OKS).

Methods

This study was done in a single centre with appropriate ethical committee approval and consent. Forty-nine patients were enrolled in the study. The scores were recorded pre-operatively and at 12 months after the interventions. Analysis was done for correlation of the outcome with patient characteristics, radiological and arthroscopic grading.

Results

Patients below the age of 56 years, with partial thickness chondral lesions, grade I-III Kellgren-Lawrence (KL) radiological grading and grade I-II Outerbridge arthroscopic grading showed significant improvement at 12 months.

Conclusion

Arthroscopic debridement is effective in younger patients with mild to moderate arthroscopic and radiologically graded osteoarthritis of the knee joint.

## Introduction

Osteoarthritis is a degenerative condition of the joints which results in debilitating pain and stiffness. Knee osteoarthritis is the most common joint pathology and the leading cause of disability in the United States of America [1]. The prevalence in India is currently estimated to be 28.7% [2] and international databases report a 113.25% rise and a three-fold increase in disease and economic burden in the last three decades [3-5]. With a population of over 1.4 billion people, this frames knee osteoarthritis as a present and worsening health crisis for the Indian population with a significant burden to both the individual and the community.

Osteoarthritis is the natural response to joint cartilage damage that occurs during repetitive wear or trauma. Injured hyaline cartilage is replaced by fibrocartilage with worse biomechanical properties leading to worsening pain and function. Clinical features of knee osteoarthritis are pain, restricted motion, crepitations, bony tenderness, bony enlargement, and instability [6]. It can be diagnosed radiographically by imaging in the antero-posterior, lateral and skyline projections to visualize the tibio-femoral and patella-femoral joints. Images are assessed for indicative changes, joint space narrowing, osteophyte formation, subchondral cysts and subchondral sclerosis. The most widely used radiographic classification is the Kellgren-Lawrence (KL) system based on antero-posterior projections of the tibio-femoral joint [7,8]. It assigns five grades based on the severity of radiographic changes from grade 0 to grade 4 [9].

The Oxford Knee Score (OKS) is a patient-reported outcome measure of the function and status of the knee joint consisting of 12 items, with good validity and reliability [10]. The maximum possible score is 48, which indicates a well-functioning joint and a minimum score of 0 which indicates severe arthritis.

The Outerbridge classification for chondral lesions was proposed after analysing the under surface of the patella in a series of patients undergoing arthroscopic meniscectomies. The lesions are graded from 1 to 4 [11]. This was then expanded to include the whole knee with good reliability and validity. This system is currently the most used system to grade chondral lesions arthroscopically [12-14].

The surgical treatment options for osteoarthritis of the knee include debridement, lavage, chondroplasty, bone marrow stimulating techniques, chondrocyte transfer, chondrocyte implantation, high tibial osteotomy (HTO), unicompartmental knee arthroplasty (UKA) and total knee arthroplasty (TKA) [15]. Arthroscopic debridement involves debridement with lavage, loose body removal, partial meniscectomy, synovectomy and chondroplasty as required. It improves symptoms by removing cartilaginous debris and inflammatory factors [16,17]. Though there is no consensus in the literature, it has been recommended for the subset of patients with mild to moderate osteoarthritis and meniscal injury [18-20]. Furthermore, favourable outcomes have been demonstrated in the short to mid-term in patients with the following characteristics - mild to moderate osteoarthritis, presence of loose bodies and/or meniscal injuries, neutral alignment of the lower limbs or minimal varus angulation, younger patients and those who have failed conservative treatment [16,17,20-24]. Despite this, its role in the treatment of knee osteoarthritis remains debatable with critics reporting it to be no more effective than a placebo [25].

Arthroscopic debridement would be a favourable management option in the Indian population as a treatment that could offer symptomatic improvement while being more cost-effective than alternatives such as total knee replacement. There have been very few prospective studies that have analysed the improvement after arthroscopic debridement, if any, to both the radiological and arthroscopic grades. Therefore, the aim of this study was to analyse the outcomes of arthroscopic debridement in a prospective case series and investigate any correlation to patient and disease characteristics, including the KL radiological grade and the Outerbridge arthroscopic classification.

## Materials and methods

This is a prospective case series of 49 patients who were treated for osteoarthritis of the knee with arthroscopic debridement. This study was registered, and approval was obtained from the ethical committee of Chalmeda Anand Rao Institute of Medical Sciences, Karimnagar in India (Research Ethics Committee certificate registration number: 18111011006D). Informed consent was obtained from all the patients. This project was done in a teaching hospital over a period of two years. The inclusion criteria were patients more than 18 years of age who had clinical symptoms and signs of osteoarthritis of the knees with radiographic grades I-IV. The exclusion criteria were patients who had other rheumatoid pathologies contributing to arthritis and any previous trauma or surgeries to the lower limb. The patients were followed up for one year after surgery. Patients with clinical and radiological features of osteoarthritis, who had failed at least two months of conservative treatment, were offered the option of arthroscopic debridement. The radiographs were graded according to the KL classification by two orthopaedic surgeons. Full-length radiographs of the lower limbs were obtained to assess for mal-alignment. Patients who opted in were enrolled in the study after they provided informed written consent. Pre-operative OKSs were recorded. The arthroscopic debridement was done by the same sub-specialty-trained orthopaedic surgeon as a day-case procedure. The Stryker Arthroscopy Endoscopy camera and arthroscopy system were used. This was done using the standard antero-medial and antero-lateral portals. The chondral defects were divided into the full thickness and partial thickness and classified according to the Outerbridge system of classification. Chondroplasties, partial meniscectomies and partial synovectomies were done in patients who required them. Six litres of normal saline was used for the lavage. The participants did not receive any intra-articular steroid injection or chondroprotective drugs or physiotherapy or any other intervention after the procedure. The patients were discharged home on the same day with instructions to fully weight bear as able with no restriction of activities. The initial follow-up was at eight weeks and a second follow-up, 12 months after surgery, when the OKS was recorded. The mean and the standard deviation (SD) for the age and Body Mass Index (BMI) of the study subjects were calculated. The results were analysed by performing univariate and multivariate correlation regression analysis for the variables and the improvements in the arthroscopic and radiological grades were tested for significance using the paired t-test, with the p-value set at 0.05 for significance. Microsoft Excel (Microsoft Corporation, Redmond, WA, USA) and IBM SPSS Version 28 (IBM, Armonk, NY, USA) was used for the analysis.

## Results

A total of 49 patients were registered for the study (n=49). There were 28 males (57%) and 21 females (43%). The average age was 57.67 years (SD=5.58). The average BMI was 24.53 (SD=2.04). Angular deformity (defined as varus or valgus angulation >10 degrees) was present in eight patients (16%). Six patients (12%) had KL grade I, 27 patients (55%) had KL grade II, 12 patients (24%) had KL grade III and four patients (8%) had grade IV (Table 1). Nine patients (18%) had full-thickness chondral defects and the remaining had partial-thickness chondral defects. Forty patients (82%) had no loose bodies, whilst they were present in nine patients (18%). Meniscal tears were present in 14 patients (29%). Arthroscopically, by the Outerbridge system of classification, there were 4 grade I knees (8%), 27 of grade II (55%), 12 of grade III (24%) and six of grade IV knees 12% (Table 2).

**Table 1:** Kellgren-Lawrence classification of the study subjects

Kellgren-Lawrence grade	Number of patients (%)
I	6 (12%)
II	27 (55%)
III	12 (24%)
IV	4 (8%)

**Table 2:** Outerbridge classification of the study subjects

Outerbridge grade	Number of patients (%)
I	4 (8%)
II	27 (55%)
III	12 (24%)
IV	6 (12%)

The overall average OKS for the 49 patients improved from 27.46 (SD=5.10) pre-operatively to 37.85 (SD=4.51) at the first follow-up in 12 months (Table 3). 

**Table 3:** Pre- and post-operative mean Oxford Knee Scores SD - Standard Deviation

Mean Oxford Knee Score pre-operatively (SD)	Mean OKS at 12 months post-operatively (SD)
27.46 (5.10)	37.85 (4.51)

In performing univariate analysis and multivariate analysis using linear regression there was a statistically significant correlation to the improvement of the OKS in patients below 56 years of age (p=0.014) and for partial thickness chondral defects (p<0.001).

There was significant improvement in patients with KL grades I (p-value=0.035), II (p-value<0.001) and III (p-value<0.0001) (Table 4) and Outerbridge grade I (p=0.0002) and II (p<0.00001) compared to the pre-operative baseline OKS (Table 5).

**Table 4:** Post-operative improvement in Oxford Knee Scores correlated statistically to the Kellgren-Lawrence subtypes

Kellgren-Lawrence grade	P-value
I	0.035
II	<0.001
III	<0.0001
IV	0.9

**Table 5:** Post-operative improvement in the Oxford Knee Scores correlated to the Outerbridge subtypes

Outerbridge grade	P-value
I	0.0002
II	<0.00001
III	1.0
IV	1.68

There were no adverse events or complications (bleeding, deep vein thrombosis, pulmonary embolism, neurovascular, soft tissue and bony injury). None of the patients at the one-year follow-up opted for TKA, UKA or HTO.

## Discussion

This case series demonstrated favourable outcomes in the mid-term for arthroscopic debridement by way of improvement in the OKS from the pre-procedure baseline with significant correlations to the age, KL grade, Outerbridge grade and chondral defects. The success rate of this procedure in patients with early osteoarthritis for whom conservative measures have failed is 70% [17]. A survey of experienced surgeons in this procedure showed good outcomes for patients with ages below 60 years and KL grades of I and II [20]. Law et al. reported improvement in patients with mild to moderate (KL I and II) disease in a series of 169 patients [21]. Moseley et al. observed no improvement but their results are debatable as an unvalidated Specific Knee Pain Scale was used [25]. A meta-analysis showed that correct patient selection leads to 60% having excellent to good outcomes and 6.1% of the patients requiring a TKA at one year [26]. None of the patients opted for TKA, UKA or HTO procedures at the one-year follow-up in our series. In the series by Steadman et al., the overall satisfactory result was 87% and patients with Outerbridge grade IV lesions had poor outcomes [27]. We did not observe any significant improvement in the OKS for patients with Outerbridge grade III and IV lesions.

Arthroscopic debridement is recommended as a temporising procedure in the mid-term after the failure of conservative measures and when the pathology does not warrant arthroplasty [21,22,24]. A systematic review and meta-analysis demonstrated significant improvement in pain, movement, and quality of life in the short term, between three and six months [26]. The United States of America has a relatively young cohort of osteoarthritic patients with more than half, below the age of 60 years [28]. In our series, patients below 56 years had significant improvement in their OKS. Other studies reported significant improvements including resting pain in the short and mid-term, using other validated instruments [29]. Patients improved in mid-term at 12 months in our series, evidenced by the improved OKSs and no conversions to other surgical procedures. Patients with mild to moderate chondral defects improve significantly after this procedure [21,23,29]. Patients with Outerbridge grade II lesions were the best responders in our series. Patients below 60 years improved more compared to older patients in the series of Mayr et al., [20]. Uthraraj et al. reported improvement in the patient outcomes recorded by the Knee Society Score in a similar demographic, in patients with early osteoarthritis, minimal varus malalignment, meniscal pathology and loose bodies in the medium term [30]. Age and early stages of osteoarthritis play an important role in predicting the outcomes of the intervention.  This strengthens the case for arthroscopic debridement as a temporising procedure for younger patients with less severe osteoarthritis.

Arthroscopic debridement for osteoarthritis is a safe and simple procedure with minimal to no inpatient stay, shorter rehabilitation and lesser post-operative pain as compared to TKA. The risk of Prosthetic Joint Infection (PJI) and the finite longevity of the implants are factors to be considered in TKA [27,31]. None of the patients in our cohort required admission to the hospital post-operatively. This can have a significant impact on the triaging of hospital beds in crisis times. BMI, varus or valgus malalignment greater than 10 degrees, the presence of loose bodies and meniscal tears were correlated with outcomes in other similar studies [20-23,32]. We did not find any significant correlation with the above patient variables in this study.

The economic burden of osteoarthritis measured as direct per patient cost is 1,442 to 21,335 United States Dollars. The higher costs are attributable to arthroplasty [33]. The Quality Adjusted Life Year (QALY) analysis for people undergoing arthroscopic debridement in Britain is below 30,000 Great British Pounds [23]. In a low-resource setting like India where this study was undertaken, this can have a sizeable impact on individual and public finances.

The commonly reported adverse events with this procedure are infection, deep vein thrombosis, pulmonary embolism and death which are very low to negligible in occurrence [21,31]. Malettis et al., from their series of 20,770 patients, reported a deep vein thrombosis risk of 0.25% and pulmonary embolism risk of 0.17% [34]. We did not have any adverse events in the peri-operative period or the course of follow-up. The low incidence of risk for adverse events makes this a safe procedure for mild to moderate osteoarthritis.

 The strengths of this study are that it was a case series where multiple patient variables were analysed for correlation including the arthroscopic Outerbridge grading, which is sparsely reported in similar cohorts. A patient-reported outcome measure tool which was validated and knee osteoarthritis specific was used to assess improvement.

The limitations of this study are that it is a single surgeon case series with a limited number of patients. There was no comparison done with a sham surgery or placebo cohort, which would have validated the results further.

## Conclusions

Arthroscopic debridement is an effective temporising procedure in the short and mid-term. It is particularly effective for younger patients, have mild to moderate radiographic and arthroscopically graded osteoarthritic lesions. It is a safe procedure that can be done as a day-case procedure. Other patient variables such as BMI, presence of loose bodies, meniscal tears, varus or valgus angulation of the joint might have a bearing on the outcomes, though it was not significant in our series.

This surgical modality is particularly an attractive option for patients in developing countries where there is no state insurance funding for health care, due to the cost of the procedure, as compared to other options and in delaying the major arthroplasty surgery.
